# Anti-inflammatory Properties of Stingless Bee Honey May Reduce the Severity of Pulmonary Manifestations in COVID-19 Infections

**DOI:** 10.21315/mjms2020.27.2.17

**Published:** 2020-04-30

**Authors:** Mohd Zulkifli Mustafa, Shazana Hilda Shamsuddin, Siti Amrah Sulaiman, Jafri Malin Abdullah

**Affiliations:** 1 Department of Neurosciences, School of Medical Sciences, Universiti Sains Malaysia, Kelantan, Malaysia; 2 Department of Pathology, School of Medical Sciences, Universiti Sains Malaysia, Kelantan, Malaysia; 3 Department of Pharmacology, School of Medical Sciences, Universiti Sains Malaysia, Kelantan, Malaysia; 4 Brain and Behaviour Cluster, School of Medical Sciences, Universiti Sains Malaysia, Kelantan, Malaysia

Dear Editor,

We are closely observing the progress of the new coronavirus global pandemic. The virus spreads rapidly, and in worst-case scenarios, it can deplete local medical resources. According to the World Health Organization (WHO), this highly contagious respiratory disease followed an exponential trajectory to arrive at 1,210,956 confirmed cases of infection (3,662 in Malaysia) with 67,594 deaths globally, as of 6 April 2020. To date, the lack of early treatment options has caused a major problem in disease management. The uncertainty of the outbreak will continue to affect the general public until the first antidote or vaccine arrives. Within this difficult context, this letter describes the evidence-based therapeutic properties of a local resource, stingless bee honey. As a healthy and functional food, it may be used to reduce the severity of pulmonary manifestations for newly-infected patients.

Coronavirus disease 2019 or officially named as COVID-19 has caused devastating social, economic and medical impacts worldwide. Originally detected in Wuhan, China (1), it was first reported to the WHO on 31 December 2019. The disease emerged as a new pandemic, leading to the most aggressive disease containment effort in human history. Mainly targeting the lungs, the virus appears to be mediated via virus-laden droplets and aerosol micro-particles (2). The virus seems transmittable via asymptomatic infection and includes prolonged virus shedding in survivors of up to 37 days (1), inducing a panic situation.

In a retrospective cohort study in Wuhan, China, published in March 2020, most patients presented with fever and cough, followed by sputum production and fatigue. Further complications include sepsis and acute respiratory distress syndrome (ARDS) with ventilator-associated pneumonia occurring in 31% of patients requiring invasive mechanical ventilation. Half of the non-survivors experienced a secondary infection, dying as a result of respiratory failure, heart failure, and/or septic shock whereby 48% of those who died had comorbidity. These patients had clearly elevated interleukin-6 (IL-6) cytokine, which increased with illness deterioration in non-survivors throughout the clinical course as compared with survivors. Viral detection appears to be sustained in the throat samples of both survivors and non-survivors (1).

Similar to Severe Acute Respiratory Syndrome (SARS) in late 2002, histopathological examination of patients who died from COVID-19 revealed diffused alveolar damage, morphological changes with varying degrees of severity, prominent hyperplasia of pulmonary epithelial cells, and activated alveolar and interstitial macrophages. These findings indicate an association between the profound pulmonary pathology observed and the intense local inflammation due to excessive host immune response (2).

It is well-known that the innate immune cells, mainly the pulmonary macrophages and dendritic cells (DC), are involved in acute inflammatory response, mediating early host defense in response to viral infection until an adaptive response can be generated (2). During a viral infection, the viral proteins are recognised by the pathogen-associated molecule patterns (PAMPs) that trigger the toll/interleukin-1 receptor-like (TIR) domain-dependent association and further recruit the IL-1 receptor-associated kinase (IRAK), subsequently inducing other factors involved in activating the transcription factor, nuclear factor kappa B (NF-κB). The activation generates the expression of several inflammatory cytokines, such as the IL-1b, IL-6, and tumour necrosis factors (TNF)-α (3). Studies indicate that the IL-6 regulates the differentiation of monocytes into macrophages and aggregates the production of B-cell IgG while affecting the negative regulation of dendritic cell maturation and promoting the Th2 response; these roles highlight IL-6’s essential contribution in the activation and regulation of the immune response against viruses (4).

One prominent Severe Acute Respiratory Syndrome-Coronavirus (SARS-CoV) study found that highly-polarised human bronchial epithelial Calu-3 cells responded to the viral infection by secreting IL-6, IL-8 and gamma interferon (IFN-γ)-inducible protein 10 (IP-10). These pro-inflammatory cytokines played a pivotal role in the production of other cytokines and phagocytosis while activating the naive T cells by modulating various functions of macrophages and DC (2). Further in-vitro verification using human peripheral blood also showed profound production of various inflammatory cytokines and confirmed that the SARS-CoV-induced Calu-3 cell cytokines were potent in stimulating macrophages and DC (2). In another coronavirus study using mouse hepatitis virus 3 (MHV3), the fixation of the spike (S) protein of MHV3 on toll-like receptor 2 (TLR2) activated the pro-inflammatory cytokines IL-6 and TNF-α production, but not viral replication (3).

Although this early epithelial response is beneficial to combatting pathogens, extreme reactions could potentially exacerbate inflammatory responses, leading to severe tissue damage (4, 2). Empirical studies support the important role of IL-6 during viral infections but numerous reports also propose that the up-regulation of IL-6 expression may have negative consequences for the cellular immune response against viruses. Here, several potential mechanisms involving IL-6 might affect viral clearance, leading to the establishment of a persistent viral state in infected hosts (4). For example, during chronic hepatitis C virus (HCV) infections, the serum IL-6 levels are associated with the viral load and the period of infection (3). Clinical studies also show exacerbation of outcomes involving viral infection in humans and animals, linking the increased systemic levels of IL-6 with the chronic occurrence of influenza and several other viruses. (4). Therefore, it is hypothesised that over-expression of IL-6 during certain viral infections potentially enhances virus endurance and/or exacerbation of disease pathogenesis (4).

Potentially useful in these cases, honey is a natural substance with several known benefits. Honey is rich in phenolic compounds, mainly flavonoids and phenolic acids, which act as potent antioxidants (5). Honey’s anti-inflammatory action is also well-documented (6). Stingless bee honey is a type of honey produced by a stingless bee (*Meliponine* sp.) (7) that is richer in polyphenols compared to other types of honey (8). Stingless bee honey is only produced in tropical and subtropical regions and features a sweet-sour taste, high moisture content and minimal crystallisation.

Recent evidence indicates that stingless bee honey has many potential therapeutic benefits, including antibacterial qualities (9), anti-obesity impacts, (10) and others (11–13). Stingless bee honey possesses several valuable properties under pathological conditions, rendering it a promising functional food that could be a reasonable dietary intervention to prevent or treat chronic subclinical systemic (CSSI) related diseases (8).

Recent research in late 2019 showed that in-vitro treatment of the lipopolysaccharide (LPS)-stimulate macrophages with stingless bee honey inhibited the TNF-α parameter by 23.0% and IL-6 secretion was reduced by 43.9%. Stingless bee honey also significantly reduced interferon secretion with inhibition of up to 88.8% (14). The findings of this study aligned with previous in-vivo research where stingless bee honey decreased the circulating levels of C-reactive protein (CRP), TNF-α, IL-1β, IL-6, IL-8 and monocyte chemoattractant protein-1 (MCP-1). It also diminished NF-κB and p38 mitogen-activated protein kinase (MAPK) signaling in different tissues of LPS-induced rats. Simultaneously, stingless bee honey boosted antioxidant defenses and showed a capability to reduce inflammation (8).

Analytical chemistry of stingless bee honey indicates several potential phenolic compounds, including gallic acid, caffeic acid, chrysin, cinnamic acid, 2-hydroxycinnamic acid, kaempferol, p-coumaric acid, catechin, quercetin-3-O-rutinosid, caffeic acid phenethyl ester and 4-hydroxybenzoic acid (8). Vitamin C was also detected at 83.34 mg/100 g stingless bee honey, much higher than Tualang honey at 29.5 mg/100 g (16).

In a clinical observation of healthy adults after honey (*Apis* sp.) consumption, results showed a potential use of honey during pathological conditions (15). A clinical trial indicated supplementing a diet with 1.5 g/kg of honey increased antioxidant levels (8). Even though the high heterogeneity of honey composition associated with geographical origin could give variable outputs in clinical settings (14), the anti-inflammatory properties seem to be related to the phenolic compound that is consistently present in stingless bee honey.

Additionally, secondary bacterial infection following viral infection occurs in 50% of COVID-19 patients (1). The consumption of stingless bee honey could again be beneficial here as stingless bee honey has shown potential antibacterial activity against Gram-positive and Gram-negative bacteria. The properties against the pathogenic bacterial result from the synergistic action of the different antimicrobial factors in honey, such as osmosis, the presence of phenolic compounds, and the production of hydrogen peroxide, all of which act against the pathogenic bacteria (9).

In summary, primary cytokines initiation is crucial in the establishment of innate immunity; however, an over-reactive inflammatory response can also exacerbate the disease without an increase in viral replication (3). Virus manipulations that lead to the overexpression of IL-6 cytokines perhaps aggravate the pathogenesis. Here, acute epithelial cytokine-mediated up-regulation of cytokine responses, in association with macrophages and DC, possibly represent a vital pathway in SARS pathogenesis (2). The inhibitory properties of honey on inflammatory signaling and its crucial role in harmonising the IL-6 cascades may be an alternative approach to limiting the disease progression of coronavirus. The pharmacological features of stingless bee honey are often related to the presence of bioactive compounds, mainly phenolic compounds (14). Therefore, it is crucial that we standardise quality control on stingless bee honey production and begin strategic design for clinical trials in pneumonia management.

Meanwhile, acknowledging the inevitability of conventional infectious disease management, the key role of alternative medicine like honey should not be dismissed in this critical moment. As mentioned in Quran surah Al-Nahl (the Bee) verse 69: “then eats from all the fruits and follows the ways of your God laid down [for you], there emerges from their bellies a drink, varying in colours, in which there is healing for people, indeed that is a sign for a people who give thought”. Thus, the stingless bee honey may be considered as a functional food to complement treatment of early infected patient during this lethal outbreak.
